# A meta-analysis of the role of neighborhood deprivation in psychotic disorders

**DOI:** 10.1007/s00127-025-02980-7

**Published:** 2025-08-06

**Authors:** Sydney H. James, Thania Galvan, Ashley Zollicoffer, Gregory P. Strauss

**Affiliations:** 1 Department of Psychology, University of Georgia, 125 Baldwin St, Athens, GA 30602 USA; 2 Department of Psychology, Louisiana State University, Baton Rouge, LA USA

**Keywords:** Psychosis, Neighborhood deprivation, Social determinants of health, Disparities

## Abstract

**Purpose:**

Although current interventions have proven effective for improving outcomes for individuals with psychotic disorders, this population continues to encounter challenges and health disparities. Recently, researchers have investigated how social determinants of health influence the incidence and outcomes of psychotic disorders. In particular, neighborhood deprivation (i.e., a composite index capturing the social, material, and environmental challenges of a particular area relative to the broader area [1]), has been investigated in numerous studies on psychotic disorders but research has yet to consolidate and quantify its significance. The current meta-analysis assesses neighborhood deprivation and its relationship to psychotic disorder incidence and symptoms.

**Methods:**

Articles published prior to April 1, 2024 were identified via two bibliographic databases: PubMed and PsycINFO. The literature search yielded 17 studies consisting of 59,719 cases for the meta-analysis investigating the relation between neighborhood deprivation and psychotic disorder incidence. Six studies of 2,790 cases were included in the meta-analysis assessing the relation between neighborhood deprivation and psychotic disorder symptoms.

**Results:**

There was a robust relation between psychotic disorder incidence and neighborhood deprivation, such that as neighborhood deprivation within an area increased so too did the incidence. There was no evidence of a substantial relationship between neighborhood deprivation and psychotic disorder symptoms.

**Conclusions:**

The results of this study identified a social determinant of health that has high relevance to the incidence of psychotic disorders. Findings underscore the need to develop multi-level interventions to address neighborhood deprivation and reduce resource inequalities across geographical locations.

**Supplementary Information:**

The online version contains supplementary material available at 10.1007/s00127-025-02980-7.

## Introduction

Psychotic disorders are among the most disabling illnesses worldwide and are associated with a variety of life-long impairments (e.g., lower life expectancy, poor physical health, and significant functional challenges) [2–5]. Recent research has highlighted the critical impact of social determinants of health (SDOH) on psychotic disorders [6–8]. The World Health Organization defines SDOH as the conditions in which people exist and that are shaped by macro-level forces (e.g., social norms, policies, and political system) [9]. In the past several decades, research examining the relation between SDOH and mental health has grown due to an enhanced global understanding of the importance of nonmedical factors on the development and manifestation of a variety of mental health conditions [10–13]. In relation to psychosis, a variety of individual SDOH (e.g., racism, lower socioeconomic status, migration status, homelessness, and social disconnection) have been associated with a greater incidence of psychotic disorders and with poorer outcomes (i.e., more severe symptoms, worse functional outcomes) in individuals with psychotic disorders [7, 14]– [15]. While these studies provide a foundational understanding of the relation between SDOH and psychotic disorders, they do not allow us to consider the cumulative effects of multiple, overlapping SDOH and how they come to influence psychotic disorders.

Utilizing a composite measure that captures a variety of SDOH may provide a more comprehensive representation of geographical SDOH and help us better predict health outcomes [16–19]. One composite measure that has been increasingly used in psychosis research is neighborhood deprivation. Neighborhood deprivation is a composite index that captures the social, material, and environmental challenges of a particular area relative to a broader area [1]. Neighborhood deprivation incorporates multiple SDOH and expands our understanding of SDOH beyond those that are most often studied (e.g., SES, urbanicity, migration) in psychosis research. Neighborhood deprivation also allows us to make cross-area comparisons to more comprehensively understand the impact of neighborhood deprivation on the incidence and course of psychosis. In fact, several countries (e.g., New Zealand, United States, United Kingdom) have indices that measure neighborhood deprivation across various geographical regions [20]. Across these indices, there are cross-country commonalities in the SDOH included while also country-specific SDOH that capture the unique circumstances for that country [20, 21]. Despite indices differences, they capture information about social and environmental inequalities present in that country, and thus, can be utilized to inform policy and programs to reduce potential inequalities across areas.

The influence of neighborhood deprivation on health has been explored in a variety of health conditions (e.g., epilepsy, cancer mortality, premature mortality), including psychotic disorders [22–25]. A systematic review exploring the relationship between psychosis and neighborhood deprivation found that deprivation was associated with greater incidence rates of psychotic disorders, such that there was an increased rate of psychotic disorders in more deprived neighborhoods [24]. Although O’Donoghue et al. [24] synthesized the literature on neighborhood deprivation and psychosis, they did not provide a quantitative estimate of the relationship between neighborhood deprivation and psychotic disorder incidence, nor did they exclude studies that utilized a single variable to represent neighborhood deprivation. Several studies have investigated the relationship between symptom severity (i.e., positive, negative, disorganized) and neighborhood deprivation in individuals with psychotic disorders and most found nonsignificant associations between neighborhood deprivation and symptom severity [26–28]. However, Bosqui et al. [29] found a significant negative association between neighborhood deprivation and negative symptoms. Given that psychotic disorders are clinically heterogeneous, understanding the relationship between neighborhood deprivation and positive, negative, and disorganized symptom dimensions can provide insight into the role of specific clinical profiles. Discerning the nature and strength of the relationship between neighborhood deprivation and psychosis can provide the evidence to support the need for multilevel and policy focused interventions.

Ethnoracial composition is another moderator of interest due to the documented ethnoracial disparities in psychotic disorder diagnoses and outcomes (i.e., symptom severity, functional outcomes) [30–33]. Specifically, studies have found individuals of minoritized ethnoracial identities are more likely to be diagnosed with schizophrenia, have more severely rated symptoms, and experience worse functional outcomes [30–34]. Considering the significant inequities individuals of minoritized ethnoracial identities encounter in various SDOH, including housing conditions, access to resources, exposure to crime and environmental hazards (e.g., pollutants, toxins) among others [35–38], researchers have recently posited that societal and structural inequities are main contributors to these ethnoracial disparities in psychotic disorders [39–41]. Thus, evaluating ethnoracial composition can provide insight into the ethnoracial disparities in psychotic disorders.

In the current study, a meta-analysis was performed to obtain a quantitative synthesis of the literature on neighborhood deprivation in psychotic disorders, with the following aims: (1) evaluate the association between deprivation and incidence of psychotic disorders, (2) evaluate the relationship between neighborhood deprivation and symptoms (i.e., positive, negative, disorganization) in individuals with psychotic disorders, and (3) examine whether race and ethnicity moderate the association between incidence of psychotic disorders and neighborhood deprivation. The quantitative nature of a meta-analysis also allows for the opportunity to investigate whether various study characteristics moderate the overall effect size estimate. Developing a better understanding of how neighborhood deprivation is related to psychotic disorders can provide insights for research and policy that foster innovative intervention strategies and reduce the costly impact of psychotic disorders.

## Method

### Search strategy and procedure

Articles published prior to April 1, 2024 were identified via two bibliographic databases: PubMed and PsycINFO. The protocol was preregistered with PROSPERO (Record ID: CRD42024530952). The search terms used consisted of (‘deprivation’ OR ‘disadvantage’ OR ‘inequality’) in combination with (‘psychosis’ OR ‘psychoses’ OR ‘psychotic’ OR ‘schizo-’). The titles and abstracts of the publications identified by the searches were independently reviewed by two assessors. Inconsistency between raters resulted in a discussion until consensus was reached. Duplicate articles were excluded. Full texts were screened by the primary author to determine whether the publication fulfilled the inclusion criteria. A secondary search of reference lists of relevant review articles was performed.

### Inclusion and exclusion criteria

Studies were included in the analysis if they: (1) provided a composite measure of neighborhood deprivation for multiple geographical areas and its association with psychotic disorder incidence (e.g., incidence rates, incidence rate ratio [IRR], odds ratio) OR its association with symptoms of psychosis (e.g., delusions, hallucinations, negative symptoms), (2) the composite measure of neighborhood deprivation included at least three variables, (3) included a sample of participants with a psychotic disorder diagnosis (e.g., schizophrenia, delusional disorder, bipolar disorder with psychosis) according to standardized diagnostic criteria, and (4) published in English as peer reviewed journal articles or dissertations. Studies were excluded if: (1) all participants were determined to be at clinical high risk for psychosis or (2) information needed to calculate the effect sizes was not available following an attempted contact with the listed corresponding author.

### Data extraction and coding

Included studies were coded for specific neighborhood deprivation index, sample characteristics (i.e., clinical diagnoses, percentage of female participants, percentage of participants with minoritized ethnoracial identities, age), time of neighborhood deprivation occurrence (e.g., time of birth, childhood, onset of psychosis), domains included in neighborhood deprivation composite, number of IRR adjustments, study duration, start year of the study, diagnostic system (e.g., ICD, DSM), number of deprivation levels, country, study design (i.e., cohort, case-control), symptom type (e.g., delusions, hallucinations, negative symptoms), and symptom severity. The following information was also extracted from included studies: sample size, publication year, incidence rate statistics (e.g., IRRs, odds ratios), incidence rate data sufficient to calculate IRRs, and correlations between symptoms and neighborhood deprivation. When the studies did not report necessary information to conduct analyses, the corresponding authors were contacted. A total of eighteen authors were contacted for missing data. Eleven authors responded, with three providing the requested information.

### Effect size calculation

For meta-analyses of studies with an association between neighborhood deprivation and incidence of psychosis, IRR was the primary variable. To estimate the association between psychotic disorder incidence and neighborhood deprivation, incidence rates, IRRs, rate ratios, risk ratios, and odds ratios were used to calculate the IRRs. In the presence of only incidence rates, IRRs were calculated according to procedure outlined by Alexander et al. [42]. Odds ratios were used in lieu of IRRs when they were an exposure odds ratio from a cohort study or obtained from a case-control study that utilized incidence rate sampling, as these yield odds ratios equivalent to IRRs [43]. In the case of studies that compared incidence rates across multiple levels of neighborhood deprivation (e.g., tertile 1 to tertile 2, tertile 1 to tertile 3), the comparison between the highest level of deprivation and lowest level of deprivation was retained for the analyses to make the effects across studies more comparable, as the number of neighborhood deprivation levels (e.g., tertiles, quintiles, etc.) differs across studies. Furthermore, studies often focus on the comparison between the highest and lowest levels of deprivation [44, 45]. When IRRs from more than one group or deprivation domain were reported, the effect sizes and standard errors were averaged in the initial analyses, as recommended by Card [46]. When studies reported both unadjusted and adjusted IRRs, the adjusted IRR was used in the analyses. All IRRs were transformed to log odds prior to aggregation. The reported mean effect size of the studies was a back transformation from log odds.

For meta-analyses of correlations between neighborhood deprivation and symptoms, Pearson’s correlation coefficients between the neighborhood deprivation measure and the symptoms were the primary variable. Estimates other than Pearson’s correlation coefficient (e.g., independent F-ratio, probability levels from significant tests) were transformed into correlation coefficients for the analyses according to procedures outlined in Card [46]. Standardized beta coefficients from univariate analyses were used in lieu of Pearson’s correlation coefficient given their equivalence [47]. Correlations were assigned weights depending on sample size and standardized using Fisher’s Z prior to aggregation. The reported mean effect size of the studies was a back transformation from Fisher’s Z. For the primary analyses, total scores from psychotic symptom measures (e.g., Brief Psychiatric Symptom Scale [BPRS], Positive and Negative Syndrome Scale [PANSS], Scale of Negative Symptoms [SANS]) were used, if reported. In the absence of a total symptom score, the individual symptom domain scores from each study were averaged to represent total symptom scores in the analyses.

### Data synthesis and analysis

For aims 1 and 2, at least 5 studies were required to compute the meta-analysis [48]. Due to the heterogeneous nature of the domains used in neighborhood deprivation measures and of psychosis spectrum disorders, random effects models were used in all analyses. Analyses were conducted using R and the R package metafor [49]. Power analysis was conducted using the R package metapower [50]. A significant *Q* statistic (*p* <.05) indicated heterogeneity, and the degree of heterogeneity was assessed with the *I*^*2*^. *I*^*2*^ values of 25% suggest “low” heterogeneity, *I*^*2*^ values of 50% indicate “medium” heterogeneity, and *I*^*2*^ values of 75% suggest “high” heterogeneity [51].

Outliers and influential effect sizes were identified by visual inspection of forest and funnel plots and calculation of Cook’s distance and the leave-one-out test statistic of the test for heterogeneity. In the presence of influential outliers, the outliers were removed, and the analyses were reconducted and reported. Publication bias was assessed using Rosenthal’s fail-safe N [52], Egger’s regression [53] and a visual examination of a funnel plot created in R. The quality of each study was assessed using the Quality Assessment Tool [54].

### Moderator analysis

In cases of significant heterogeneity, potential moderators of effect size were examined using meta-regression. For these analyses, at least 5 effects were required per moderator. For meta-regression of IRRs, a priori variables were selected as possible sources of heterogeneity, including country, neighborhood deprivation index, time of neighborhood deprivation occurrence (e.g., time of birth, childhood, onset of psychosis), clinical diagnosis, type of IRR (i.e., unadjusted, adjusted), percentage of female participants, age of the participants, scores on the Quality Assessment Tool [54], and percentage of participants with minoritized ethnoracial identities. However, due to limited variance in the variables across studies, meta-regressions were not conducted with country, neighborhood deprivation index, and time of neighborhood deprivation occurrence (e.g., birth, onset of psychosis) as moderators. Instead, the number of IRR adjustments, diagnostic system (e.g., ICD, DSM), study duration, start year of the study, and number of deprivation levels were explored as moderators. Diagnostic system and clinical diagnosis were entered as categorical variables, whereas scores on the Quality Assessment Tool, age of the participants, percentage of female participants, percentage of participants with minoritized ethnoracial identities, number of IRR adjustments, study duration, start year of the study, and number of deprivation levels were entered as continuous variables. Due to limitations of the ethnoracial data reported and obtained, the percentage of migrants in two samples was substituted for the percentage of participants with ethnoracial identities in the sample when it was not reported. For studies that reported the number of participants in the sample that fall within a specified age range, the median age was calculated using the midpoint age of each range and the cumulative frequency. For the meta-regression of correlational effects, country, neighborhood deprivation index, time of neighborhood deprivation occurrence, and scores of the Quality Assessment Tool were selected a priori as moderators.

## Results

### Study selection

**Fig. 1:**
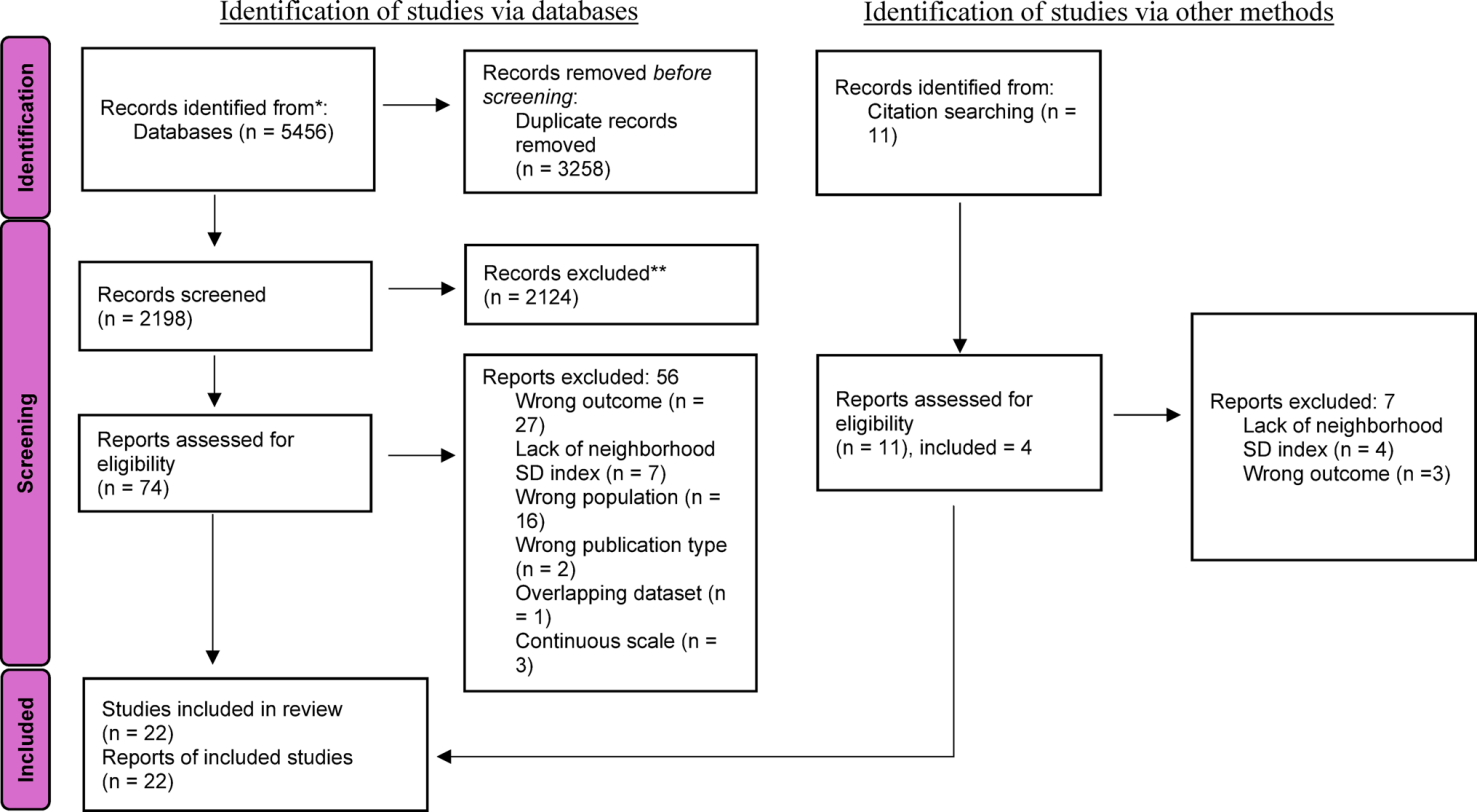
Flow diagram of study selection

A flow diagram depicting study selection, based on Preferred Reporting Items for Systematic Reviews and Meta-Analyses (PRISMA) guidelines, is presented in Fig. 1 [55]. 5,456 records were identified by the database search, as well as 11 records through citation searching. Most eligible studies utilized a categorical deprivation scale; however, three studies used a continuous scale of deprivation and, thus, were excluded. The number of studies included in the final analyses was 22. Due to the limited number of studies reporting neighborhood deprivation and symptoms, analyses of different symptom domains (e.g., delusions, hallucinations, negative symptoms) and other potential moderators (e.g., age, gender, ethnoracial composition of the sample) were not appropriate.

### Study characteristics

A total of 17 studies evaluating the association between neighborhood deprivation and incidence of psychotic disorders were included. 1 study reported on two different samples, so 18 individual samples were included. Within the 18 samples included, there were a total of 59, 719 incidence cases of psychotic disorders. Most studies included in the analyses assessed neighborhood deprivation at the time of service entry with two studies assessing neighborhood deprivation at different times (i.e., 1 study assessed neighborhood deprivation at birth and one study assessed neighborhood deprivation at the time of study entry).

A total of 6 studies examined the association between neighborhood deprivation and symptoms in individuals with psychotic disorders; all of which were included in the analysis. Within the 6 study samples, there was a total of 2,790 participants. Fordham et al. [56] was included in both meta-analyses.

The demographics reported for each study sample varied across the studies included, in that 18 studies reported age (i.e., number of cases within different age ranges; median age; mean age), 19 studies reported percentage of males and females in the sample, and 10 studies reported percentage of participants with minoritized ethnoracial identities in the sample. Details of each study are included in Table 1. IRR estimates and adjustments of each study are presented in Online Resource 1 (Table S1).

**Table 1:** Study characteristics

Study	*N*	Country	% Female	Age	% Minoritized^a^	Diagnoses	Neighborhood deprivation composite	Domains included in neighborhood deprivation composite
Kirkbride et al. [76]	218	England	42.7	NR	74.7	ICD10 F20-F29	Index of multiple deprivation	Unemployment, education, health, barriers to housing and services, living unit environment, crime
Eaton et al. [59]	722	Australia	40.6	M = 19.5	NR	DSM-IV 295.xx, 298.8, 298.9, 296.x4, 292.xx	Index of relative socio-economic disadvantage	Unemployment, occupational status, living unit environment, education, resource limitations, disability, familial structure, language barriers
O’Donoghue et al. [77]	292	Ireland	43.5	MD = 31.4	NR	DSM-IV 295.xx, 297.xx, 292.xx, 296.x4, 298.9, 298.8	Haase-Pratschke deprivation index	Recent increases in population, education, occupational status, familial structure, unemployment, living unit environment
Anderson et al. [78]	447	Canada	29.2	MD = 20	NR	ICD10 F20, F22, F25 ICD9 295, 297	Self-made	Unemployment, education, income, familial structure, living environment structure
Kirkbride et al. [61]	687	England	33.2	MD = 21^b^	25.2	ICD10 F10-F33	Index of multiple deprivation	Unemployment, education, health, living unit environment
Omer et al. [79]	255	Ireland	43.5	MD = 32	0	DSM-IV 295.x, 296.x4	Material deprivation index	Unemployment, social class, type of house tenure, car ownership
Sutterland et al. [80]	293	Netherlands	38.2	M = 31.6	NR	DSM-IV 295.xx, 297.1, 298.8, 298.9	Deprivation index	Income, welfare status, housing density, migrant density
Harrison et al. [44]	82	England	39.0	MD = 25.5	24.4	ICD10 F20-F33, F1x	Nottingham index of need	Income, housing, unemployment, crime
Boydell et al. [81]	222	England	43.2	M = 35.4	57	ICD9 295, 295.6, 296, 296.2, 296.4, 297, 298, 292.1 ICD10 F20; F25, F22, F30, F31.3, F31.2, F31.6, 28, 29, 12.5, 16.6, 19.5, 16.75, 19.75	Index of local conditions	Unemployment, overcrowding, child poverty, lack of amenities, income, education, lack of a car
Rotenberg et al. [82]	25,464	Canada	38.5	M = 28	NR	ICD9 295.x, ICD10 F20 or F25, DSM-IV 295.x	Ontario marginalization index	Unemployment, Income, living unit environment, familial structure, government payment assistance status
Lasalvia et al. [83]	558	Italy	48.7	MD = 34.5^b^	22.8	ICD10 F1x.4; F1x.5, F1x.7, F20–29, F30.2, F31.2, F31.5, F31.6, F32.3, F33.3	Self-made	Occupational status, education, familial structure, unemployment, housing status (rented vs. owned)
Veling et al. [62]	611	Netherlands	29.5	MD = 22^b^	57.9	DSM-IV 295.xx, 297.xx, 298.8, 298.9, 296.x4	Self-made	Socioeconomic level, residential mobility, ethnic diversity, living structure, crime, voter turnout, population density
Hardoon et al. [84]	7364	United Kingdom	NR	NR	NR	Schizophrenia, non-organic psychosis^c^	Townsend	Unemployment, overcrowding, resource ownership
Tortelli et al. [45]	291	France	34.7	MD = 34.3^b^	35.7	DSM-IV-TR 295.x, 297.x, 298.x, 296.x4	Priority neighborhoods	Unemployment, low-income households, social housing, familial structure
Driessen et al. [85]	186	Netherlands	NR	NR	NR	ICD9 295.x, 297.x	Self-made	Government payment assistance status, rate of migration, familial structure, migrant density, non-voter density
Fordham et al. [56]	1574	Australia	30.4	M = 19.8	7.3	First-episode psychosis (specifics not reported)	Index of relative socio-economic disadvantage	Income, education, employment, occupation, housing
Lee et al. [86] (Sample 1)^3^	8569	Wales	NR	NR	NR	ICD10 F20, F20.x, F25.x, F22.x, F24	Welsh index of multiple deprivation	Income, housing, access to services, community safety, education, health, employment, physical environment
Lee et al. [86] (Sample 2)	11,884	Wales	NR	NR	NR	ICD10 F20, F20.x, F25.x, F22.x, F24	Welsh index of multiple deprivation	Income, housing, access to services, community safety, education, health, employment, physical environment
Tibber et al. [87]	319	England	34.2	MD = 24.16	NR	DSM-IV 295.xx, 297.1, 298.8, 296.24, 296.34, 296.0x, 296.5x, 296.4x, 296.6x, 296.7	Index of multiple deprivation	Unemployment, education, health, barriers to housing and services, living unit environment, crime
Bosqui et al. [29]	223	Ireland	37.2	M = 33.09	NR	ICD10 psychotic disorder	Multiple deprivation measure	Income, employment, health and disability, education, skills and training, proximity to services, crime and disorder, and living environment
Izquierdo et al. [27]	137	Spain	33.6	M = 24.62	NR	DSM-IV 295.xx, 297.1, 296.x4, 298.8, 298.9	Mandrid City council neighborhood vulnerability	Income, unemployment, living environment, housing, immigrant proportion, life expectancy
Veru-Lesmes et al. [28]	202	Canada	29.7	M = 23.40	NR	DSM-IV first episode of non-affective or affective psychosis	Institut National de Santé Publique du Québec” (INSPQ)	Income, education, employment, familial structure, living environment
Oher et al. [26]	335	England	43.3	MD = 29	58.2	ICD10 F10-F33	Index of multiple deprivation	Unemployment, education, health, barriers to housing and services, living unit environment, crime

IRR = incidence rate ratio. M = mean. MD = median. NR = not reported. DSM = Diagnostic and Statistical Manual of Mental Disorders. ICD = International Classification of Diseases. CBS = Maastricht Statistics Department and Statistics Netherlands

^a^% Minoritized is the percentage of the sample with minoritized ethnoracial identities

^b^Median age was calculated from age-range frequency tables

^c^Diagnoses based on the Read code system (Chisholm, 1990)

### Association between incidence of psychotic disorders and neighborhood deprivation

#### Meta-analyses

**Fig. 2:**
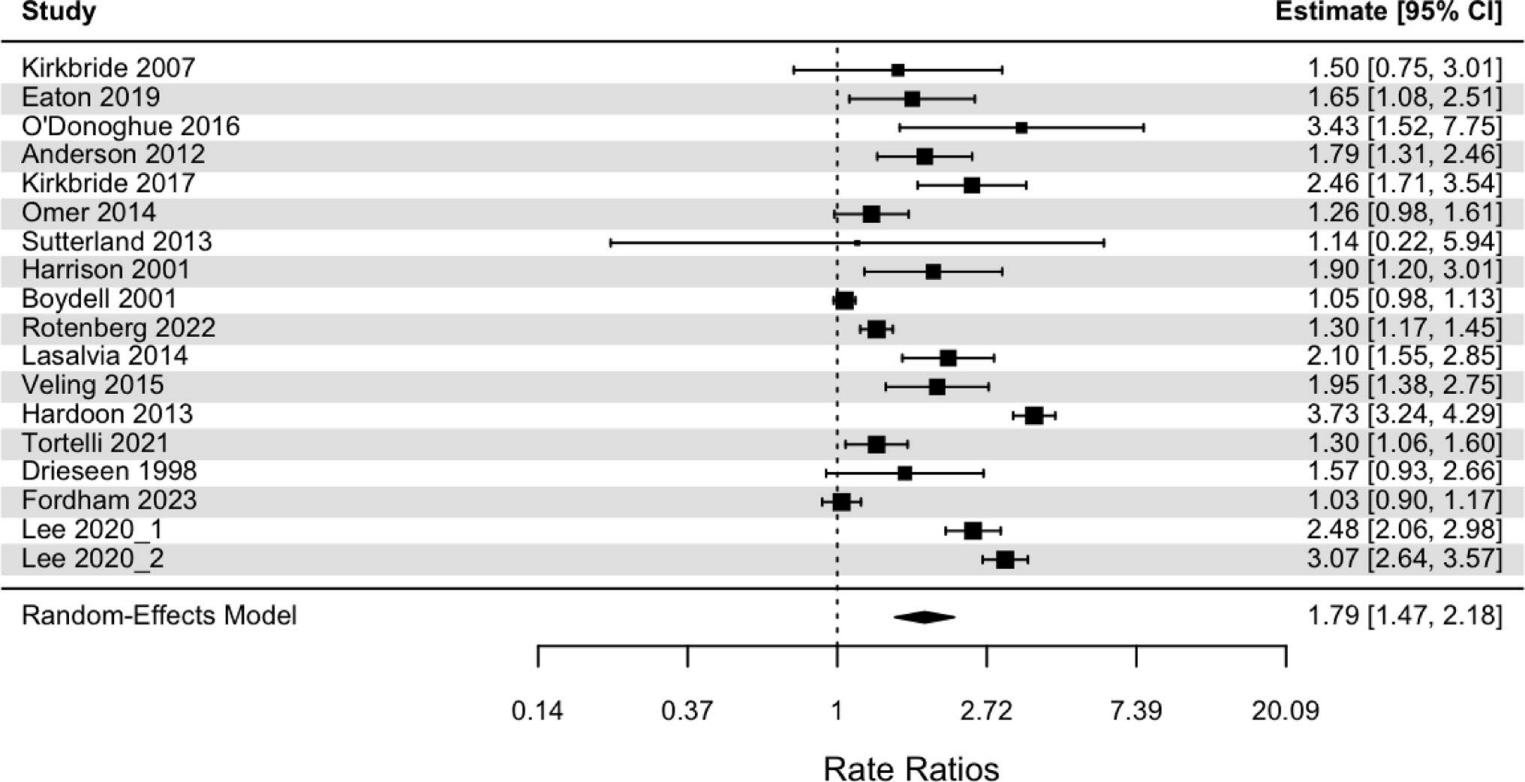
Forest plot for meta-analyses of the association between neighborhood deprivation and incidence of psychotic disorders

An initial random effects model was conducted across 18 effects. In the pooled analysis of 18 effects, there was a significant effect of neighborhood deprivation on incidence of psychotic disorders (IRR = 1.79, 95% CI [1.47, 2.18], *p* <.0001), such that there was a higher incidence of psychotic disorders in areas of greater neighborhood deprivation. There was evidence for significant high heterogeneity between effects (*Q* = 431.14, *p* <.0001; *I*^*2*^ = 93.63%). The forest plot for this initial model is displayed in Fig. 2. The funnel plot for this initial model displayed in Online Resource 1 (Figure S1) depicts abnormalities in the funnel shape and a lack of complete symmetry, suggesting potential publication bias [53]. The fail-safe number of effects was 2431, greater than Rosenthal’s threshold (5k + 10), and Egger’s regression test was nonsignificant (*z* = 0.30, *p* =.764), indicating a lack of publication bias. No influential outliers were identified. Considering significant heterogeneity and the variation in IRR estimates (adjusted vs. unadjusted) provided, a random effects model with only adjusted estimates was conducted. In the pooled analysis of 14 adjusted effects, the significant association between incidence of psychotic disorders and neighborhood deprivation remained (IRR = 1.86, 95% CI [1.49, 2.33], *p* <.0001) with continued high significant heterogeneity (*Q* = 390.30, *p* <.0001, *I*^*2*^ = 94.25%). To determine the pooled effect of unadjusted estimates, a random effects model with 16 unadjusted effects was conducted. It identified a significant association between incidence of psychotic disorders and neighborhood deprivation (IRR = 1.83, 95% CI [1.47, 2.27], *p* <.0001) that also yielded high significant heterogeneity (*Q* = 186.81, *p* <.0001, *I*^*2*^ = 94.12%). There was a nonsignificant difference in the effect sizes calculated from the model with only adjusted effects and the model with only unadjusted effects, (δ = 0.02, *SE*_*δ*_ *=* 0.16, *z* = 0.12, *p* =.902).

#### Moderation analyses

To evaluate the effects of demographic variables (i.e., age, percentage of female participants, percentage of participants with minoritized ethnoracial identities) on the relationship between neighborhood deprivation and psychotic disorder incidence, meta-regressions were conducted with each demographic variable separately to maximize the number of studies included in the model. All the effect sizes included in the meta-regressions with age and percentage of female participants had to be crude IRRs, as the adjusted IRRs accounted for age and gender. Thus, 6 effect sizes were evaluated in the meta-regression with median age, 12 effect sizes were evaluated in the meta-regression with percentage of female participants, and 9 effect sizes were analyzed in the meta-regression with percentage of participants with minoritized ethnoracial identities. Analyses found a nonsignificant effect of percentage of female participants (*Q*_*M*_ = 0.06, *p* =.803, *R*^*2*^ = 0), percentage of participants with minoritized ethnoracial identities (*Q*_*M*_ = 0.07, *p* =.798, *R*^*2*^ = 0), and median age (*Q*_*M* =_ 0.16, *p* =.685, *R*^2^ = 0) on the relationship between neighborhood deprivation and psychotic disorder incidence.

To evaluate whether the number of adjustments to the IRR accounts for significant heterogeneity present in the initial random effects model, moderation analysis was conducted. In a meta-regression analysis of the 18 effects, the number of IRR adjustments was nonsignificant, (*Q*_*M*_ = 1.97, *p* =.160, *R*^*2*^ = 0.09). The number of IRR adjustments accounted for 9% variance in the model.

Moderation analysis was conducted to determine whether the significant heterogeneity could be explained by diagnostic category and diagnostic system (e.g., ICD, DSM). Due to limited effect sizes for each diagnostic category, effect size estimates were compared between nonaffective psychosis (k = 12) and affective psychosis (k = 4). Diagnostic category did not moderate the effect size (*Q*_*M*_ = 0.77, *p* =.382, *R*^*2*^ = 0) nor did it explain any variance between effect sizes. For the moderation analysis with the diagnostic system, effect size estimates were compared between ICD (k = 7) and DSM (k = 7). Diagnostic system did not moderate the effect size (*Q*_*M*_ = 0.70, *p* =.401, *R*^*2*^ = 0.02).

Meta-regressions were conducted to determine the effect of study quality, study duration, and study start year on the relationship between incidence and neighborhood deprivation. Analyses found a nonsignificant effect of study quality (*Q*_*M*_ = 0.51, *p* =.475, *R*^*2*^ = 0), study duration (*Q*_*M*_ = 0.12, *p* =.726, *R*^*2*^ = 0), and study start year (*Q*_*M*_ = 0.18, *p* =.668, *R*^*2*^ = 0). Study quality, study duration, and study start year did not explain any variance in the model.

A meta-regression was conducted to determine the effect of number of deprivation levels (e.g., tertiles, quartiles, quintiles) on the relationship between incidence and neighborhood deprivation. In the meta-regression analysis, the number of deprivation levels was approaching significance (*Q*_*M*_ = 3.82, *p* =.051, *R*^*2*^ = 0.18). The number of IRR adjustments accounted for 18% variance in the model.

#### Power analysis

Considering the high amount of heterogeneity found in the initial random effects model (*I*^*2*^ = 94%), the power analysis for the random effects model is reported. The results of the power analysis suggest that, with a random-effects model, the current study has a 90% probability of detecting a small effect size (IRR = 1.68 [57]) if it exists.

### Correlations between symptoms and neighborhood deprivation

#### Meta-analyses

**Fig. 3:**
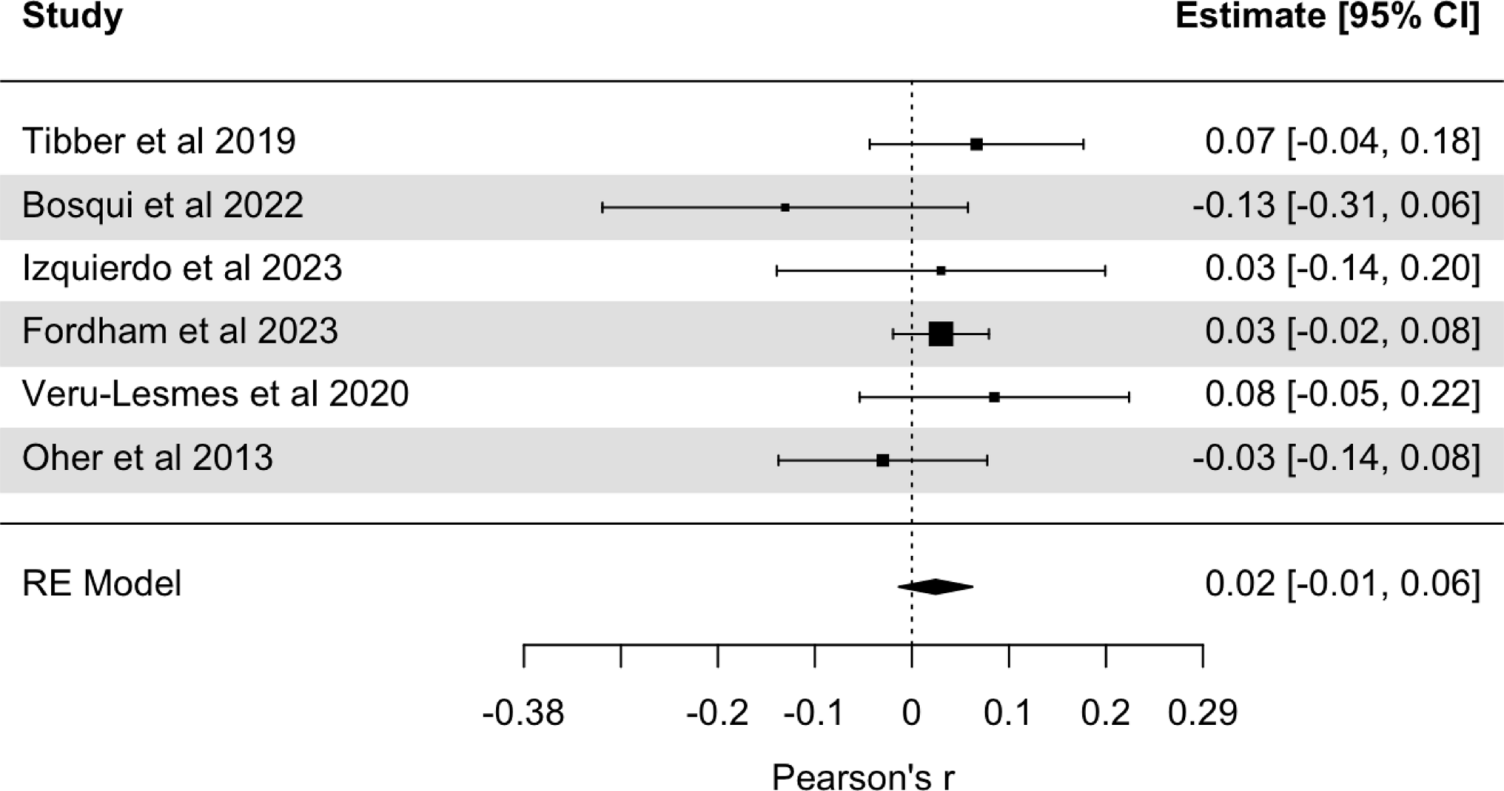
Forest plot for meta-analyses of the correlation between neighborhood deprivation and symptoms

A random-effects model was conducted across 6 effects, revealing a nonsignificant association between symptom severity and neighborhood deprivation (*r* =.02, 95% CI [−0.01, 0.06], *p* =.207). There was no evidence for significant heterogeneity between effects (*Q* = 4.93, *p* =.424, *I*^*2*^ = 0%). The forest and funnel plots for the model are displayed in Fig. 3 and Online Resource 1 (Figure S2). Egger’s regression test was nonsignificant (*z* = −0.63, *p* =.531), and the fail-safe number of effects was zero, indicating a lack of publication bias. Given the lack of significant heterogeneity between effects, moderation analysis was not conducted. The individual effect sizes for each study are displayed in Online Resource 1 (Table S2). The results of the power analysis suggest that, under a random-effects model with low heterogeneity, the current study has a 99% probably of detecting a small effect size (0.2) if it exists.

## Discussion

The current meta-analysis sought to quantify the association between neighborhood deprivation and (1) incidence of psychotic disorders; (2) the severity of positive, negative, and disorganized symptom dimensions; (3) minoritized ethnoracial identities. Several key findings emerged.

First, there was a positive association between neighborhood deprivation and incidence of psychotic disorders. Geographical areas with greater neighborhood deprivation were associated with a 79% increase in rates of psychotic disorders (IRR = 1.79, 95% CI [1.47, 2.18], *p* <.0001). The relationship between neighborhood deprivation and psychotic disorder incidence remained significant regardless of type of IRR and removal of influential outliers. Furthermore, despite the individualized nature of neighborhood deprivation composites to a particular society, these findings were consistent across countries. This suggests that, regardless of the domains included in the measure, there is inequality, resulting in neighborhood deprivation, in all countries that leads to increased psychotic disorder incidence.

Second, there was no evidence for a differential association between neighborhood deprivation and the three primary symptom dimensions in psychotic disorders (positive, negative, disorganized). This suggests that while neighborhood deprivation increases risk for developing a psychotic disorder diagnosis generally, it may not confer greater likelihood of developing a certain clinical profile characterized by specific aspects of psychopathology. Although power analysis indicated adequate power to observe a small effect, this null result should be interpreted with caution since it was based on six studies.

Third, moderator analyses yielded no significant moderators. However, the number of deprivation levels was approaching significance (*p* =.051), such that as the number of deprivation levels increases, the association between neighborhood deprivation and psychotic disorder incidence increases. This may indicate that more inequality (i.e., larger discrepancies in neighborhood deprivation) within an area increases the risk of psychotic disorders, yet more research is needed. The other nonsignificant results are likely a result of limited power and reporting inconsistencies. Importantly, the percentage of participants with minoritized ethnoracial identities did not significantly impact the relationship between psychotic disorder incidence and neighborhood deprivation. Given past research indicating that residential conditions inform ethnoracial health disparities and the well-documented ethnoracial disparity in psychotic disorder diagnoses, the results of the current study are surprising [30, 58]. However, this lack of significant moderation effect is supported by a number of studies finding that the association between psychotic disorder incidence and neighborhood deprivation remained after adjustments for race and ethnicity were made [59–62]. Thus, regardless of an individual’s ethnoracial identity, residing in socially deprived area increases the risk of developing a psychotic disorder. This may suggest that living in a socially deprived area may exacerbate the risk of psychotic disorders in individuals with minoritized ethnoracial identities. Future research could benefit from comparing the association between psychotic disorder incidence and neighborhood deprivation between different ethnoracial groups, as studies have demonstrated the larger impact of SDOH on minoritized communities [13, 63]– [64].

Certain limitations should be noted. First, there were a limited number of studies exploring the relationship between neighborhood deprivation and psychotic disorders, especially those that utilized a composite measure of neighborhood deprivation. The limited number of studies significantly reduced the statistical power, as indicated by the power analysis, and hindered the ability to conduct additional analyses on these effects. Second, inconsistencies in how data was reported across studies impacted which variables could be evaluated in moderator analyses and how much power was available to observe effects. For example, the ethnoracial composition samples was infrequently reported. Limited reporting of ethnoracial identity prompted us to use migrant status as a proxy for race and ethnicity in two studies. Although migrant status overlaps with race and ethnicity [65], it is not equivalent, and this may have contributed to the null results. Third, the current study was unable to evaluate the effect of time of neighborhood deprivation occurrence (e.g., birth, onset of psychosis) due to the restricted variance among the samples. This is an important avenue to pursue in future studies considering the relationship between obstetric complications and psychosis as well as the associations between adverse perinatal outcomes and neighborhood deprivation [66–68]. Past studies have reported that women with minoritized ethnoracial identities are more likely to experience obstetric complications [69–72], and ergo, exploring neighborhood deprivation at birth may provide insight into the disparities seen in the diagnosis of psychotic disorders. Lastly, the current study was unable to determine the impact of various IRR adjustments on the overall effect size due to the heterogeneity in the variables adjusted for in the estimates. To potentially circumvent this limitation, future studies investigating neighborhood deprivation and psychotic disorder incidence could report both unadjusted and adjusted IRR estimates.

Despite these limitations, the current study consistently found that psychotic disorder incidence is greater in areas with more neighborhood deprivation, identifying a SDOH that could be targeted via structural interventions. Structural interventions may be beneficial for providing and connecting individuals to resources needed to obtain health care, social support, and recreational/goal-directed activities. Examples of such interventions might include community outreach events, housing voucher programs, and improved public transit and pedestrian transportation systems [73]. When coupled with pharmacotherapy and psychosocial treatments, these interventions may be useful for reducing incidence of psychotic disorders and symptom severity. Additionally, new tools for mapping SDOH may be utilized to help clinicians assess the potential impact of neighborhood deprivation on an individual’s symptom profile or risk of developing psychosis. Two tools clinicians may consider using are the Structural Vulnerability Assessment Tool [74] and the Mapping Vulnerability and Privilege Exercise [75]. These tools can facilitate discussions about SDOH and potential strategies that may mitigate the impact of such factors on the client and their well-being. When these assessments are integrated with structural interventions, psychosocial treatments, and pharmacological treatment for psychosis, they may allow for stratified intervention and prevention strategies, rather than a one-size-fits-all approach.

## Supplementary Information

Below is the link to the electronic supplementary material.

Supplementary Material 1

## Data Availability

No datasets were generated or analysed during the current study.
