# Supplementary material for: A meta-analysis of the role of neighborhood deprivation in psychotic disorders
Source: Soc Psychiatry Psychiatr Epidemiol. 2025 Aug 6;60(12):2721–33. doi: 10.1007/s00127-025-02980-7 (PMC12594673; doi:10.1007/s00127-025-02980-7)
Supplement: Supplementary file 1 — Supplementary Material 1 [file 127_2025_2980_MOESM1_ESM.docx]

**SUPPLEMENTAL MATERIALS for**

**A META-ANALYSIS OF THE ROLE OF NEIGHBORHOOD DEPRIVATION IN PSYCHOTIC DISORDERS**

Sydney H. James, M.S. ^1^*

Thania Galvan, Ph.D.^1^

Ashley Zollicoffer, M.A.^2^

Gregory P. Strauss, Ph.D.^1^

1. Department of Psychology, University of Georgia, Athens, GA, USA
2. Department of Psychology, Louisiana State University, Baton Rouge, LA, USA

*Correspondence concerning this article should be addressed to Sydney James, M.S., Email: Sydney.howie@uga.edu. University of Georgia, Department of Psychology, 125 Baldwin St., Athens, GA 30602.

| **Table S1.**  *Studies included in quantitative synthesis of Psychotic Disorder Incidence* | | | |
| --- | --- | --- | --- |
| Study | Unadjusted IRR [95% CI] | Adjusted IRR  [95% CI] | IRR Adjustments |
| Kirkbride et al. [1] | 1.76 [1.02, 3.04] | 1.50 [0.74, 3.01] | Age, gender, ethnicity |
| Eaton et al. [2] | NR | 1.65 [1.08, 2.51] | Age, gender, migrant status |
| O’Donoghue et al. [3] | 3.44 [1.51, 7.84]^2^ | 3.43 [1.24, 7.75] | Age |
| Anderson et al. [4] | 1.73 [1.26, 2.36]^1,2^ | 1.79 [1.31, 2.46]^1^ | Age, gender |
| Kirkbride et al. [5] | 2.49 [2.21, 2.81] | 2.46 [1.71, 3.54]^1^ | Age, gender, ethnicity, SES, neighborhood population density, early intervention psychosis service |
| Omer et al. [6] | 1.36 [0.86, 2.14] | 1.26 [0.98, 1.61]^1^ | Age |
| Sutterland et al. [7] | 1.14 [0.22, 5.94]^2^ | NR | None |
| Harrison et al. [8] | 1.90 [1, 3.70] | NR | None |
| Boydell et al. [9] | 1.06 [1.01, 1.11] | 1.05 [0.98, 1.13] | Age, gender, ethnic minority |
| Rotenberg et al. [10] | 1.53 [1.41, 1.66]^2^ | 1.30 [1.16, 1.45] | Age, gender |
| Lasalvia et al. [11] | 2.10 [1.55, 2.85]^2^ | NR | None |
| Veling et al. [12] | NR | 1.95 [1.38, 2.75] | Age, gender, ethnicity, marital status |
| Hardoon et al. [13] | 4.65 [1.46, 7.85]^1,2^ | 3.84 [3.31, 4.46]^1^ | Age, gender, urbanicity |
| Tortelli et al. [14] | 1.33 [1.06, 1.67]^2^ | 1.30 [1, 1.60] | Age, gender |
| Driessen et al. [15] | 1.59 [0.93, 2.72] | 1.57 [0.93, 2.66]^1^ | Age, gender |
| Fordham et al. [16] | 1.03 [0.90, 1.17] | NR | None |
| Lee et al. [17] (Sample 1) | 2.65 [2.48, 2.84]^2^ | 2.48 [2.06, 2.98] | Age, gender, urbanicity |
| Lee et al. [17] (Sample 2) | 3.14 [2.95, 3.35]^2^ | 3.07 [2.64, 3.57] | Age, gender, urbanicity |
| *Note.* IRR = Incidence rate ratio. CI = confidence interval, NR = not reported. SES = socioeconomic status.  1. Averaged across groups.  2. Calculated from incidence rates. | | | |

| **Table S2.**  *Studies included in quantitative synthesis of symptom correlations* | | | |
| --- | --- | --- | --- |
| Study | N | Symptom Scale | Pearson’s *r* |
| Tibber et al. [18] | 319 | SANS, SAPS | .07^1^ |
| Bosqui et al. [19] | 223 | BPRS | -.13^2^ |
| Izquierdo et al. [20] | 137 | PANSS | .03 |
| Fordham et al. [16] | 1574 | BPRS | .03 |
| Veru-Lesmes et al. [21] | 202 | SANS | .09^2^ |
| Oher et al. [22] | 335 | SCAN Item Group Checklist | -.03^1^ |
| *Note.* SANS = Scale for the Assessment of Negative Symptoms. SAPS = Scale for the Assessment of Positive Symptoms. BPRS = Brief Psychiatric Rating Scale. PANSS = Positive and Negative Syndrome Scale. SCAN = Schedules for Clinical Assessment in Neuropsychiatry.  1. Averaged across symptoms.  2. Averaged across groups. | | | |

**Figure S1.**

*Funnel plot for meta-analyses of the association between neighborhood deprivation and incidence of psychotic disorders*


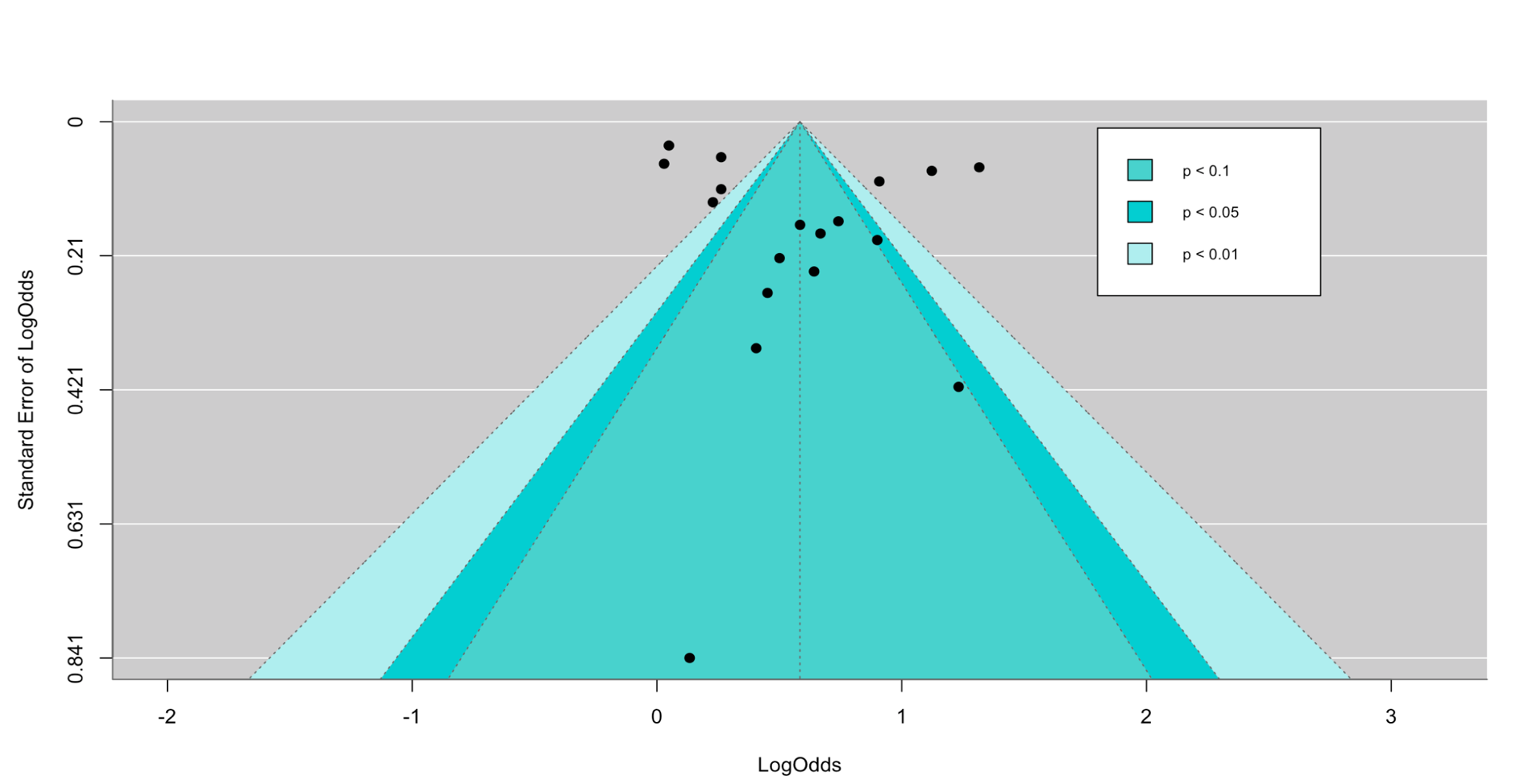


*Note.* Egger’s test statistic: z = 0.30, p = .764; Fail-safe N = 2431.

**Figure S2.**

*Funnel plot for meta-analyses of the correlation between neighborhood deprivation and symptoms*

*
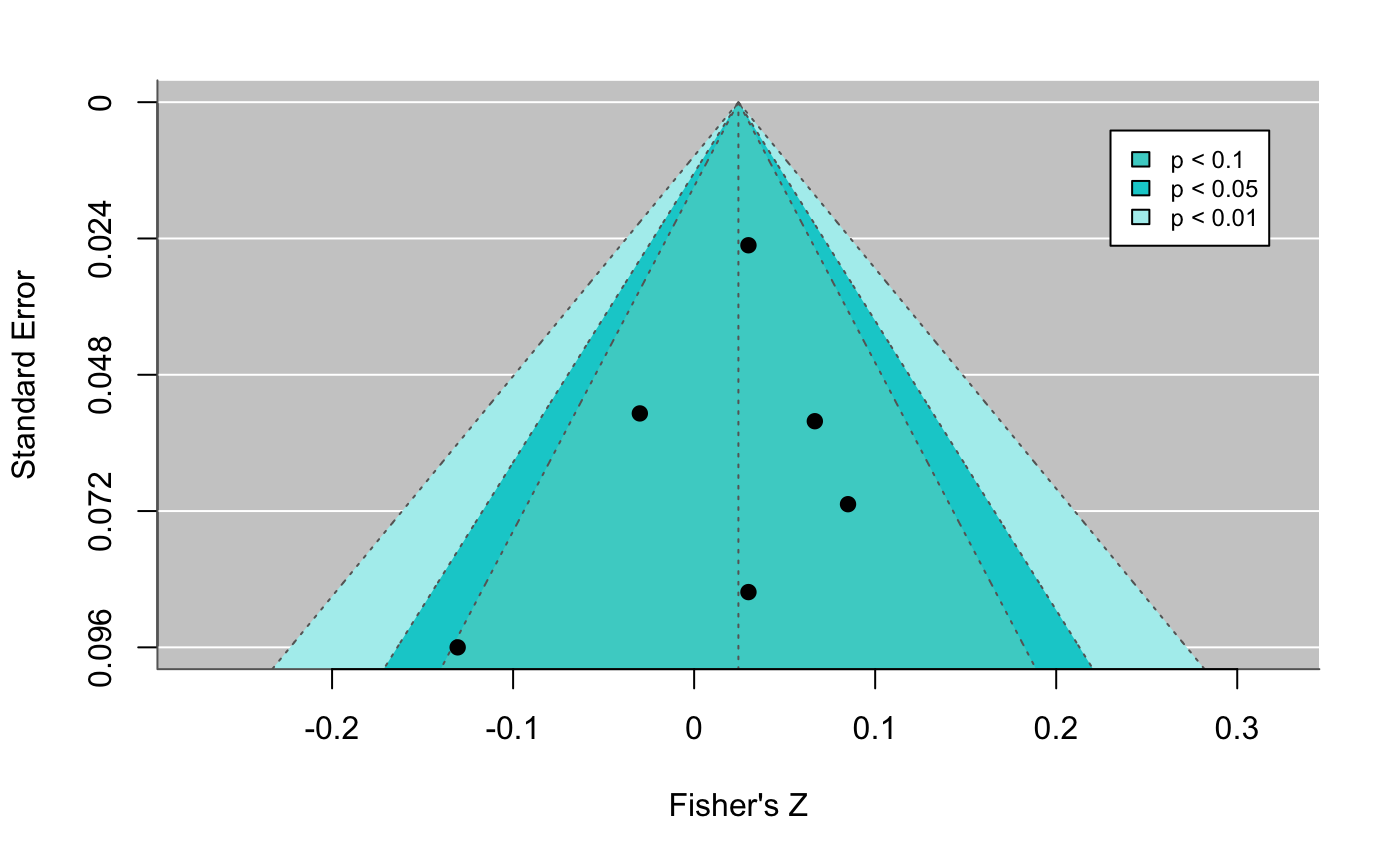
*

*Note.* Egger’s test statistic: *z* = -0.63, *p* = .531; Fail-safe N = 0.

**References**

1. Kirkbride JB, Morgan C, Fearon P, Dazzan P, Murray RM, Jones PB (2007) Neighbourhood-level effects on psychoses: re-examining the role of context. Psychol Med 37(10):1413-25.
2. Eaton S, Harrap B, Downey L, Thien K, Bowtell M, Bardell-Williams M, Ratheesh A, McGorry P, O'Donoghue B (2019) Incidence of treated first episode psychosis from an Australian early intervention service and its association with neighbourhood characteristics. Schizophr Res 209:206-11.
3. O’Donoghue B, Lyne JP, Renwick L, Lane A, Madigan K, Staines A, O'callaghan E, Clarke M (2016) Neighbourhood characteristics and the incidence of first-episode psychosis and duration of untreated psychosis. Psychol Med 46(7):1367-78.
4. Anderson KK, Fuhrer R, Abrahamowicz M, Malla AK (2012) The incidence of first-episode schizophrenia-spectrum psychosis in adolescents and young adults in Montreal: an estimate from an administrative claims database. Can J Psychiatry 57(10):626-33.
5. Kirkbride JB, Hameed Y, Ankireddypalli G, Ioannidis K, Crane CM, Nasir M, Kabacs N, Metastasio A, Jenkins O, Espandian A, Spyridi S (2017) The epidemiology of first-episode psychosis in early intervention in psychosis services: findings from the social epidemiology of psychoses in East Anglia [SEPEA] study. Am J Psychiatry 174(2):143-53.
6. Omer S, Kirkbride JB, Pringle DG, Russell V, O'Callaghan E, Waddington JL (2014) Neighbourhood-level socio-environmental factors and incidence of first episode psychosis by place at onset in rural Ireland: The Cavan–Monaghan First Episode Psychosis Study [CAMFEPS]. Schizophr Res 152(1):152-7.
7. Sutterland AL, Dieleman J, Storosum JG, Voordouw BA, Kroon J, Veldhuis J, Denys DA, de Haan L, Sturkenboom MC (2013) Annual incidence rate of schizophrenia and schizophrenia spectrum disorders in a longitudinal population-based cohort study. Soc Psychiatry Psychiatr Epidemiol 48:1357-65.
8. Harrison G, Gunnell D, Glazebrook C, Page K, Kwiecinski R (2001) Association between schizophrenia and social inequality at birth: case–control study. Br J Psychiatry 179(4):346-50.
9. Boydell J, Van Os J, McKenzie K, Allardyce J, Goel R, McCreadie RG, Murray RM (2001) Incidence of schizophrenia in ethnic minorities in London: ecological study into interactions with environment. BMJ 323(7325):1336.
10. Rotenberg M, Tuck A, Anderson KK, McKenzie K (2022) The incidence of psychotic disorders and area-level marginalization in Ontario, Canada: a population-based retrospective cohort study. Can J Psychiatry 67(3):216-25.
11. Lasalvia A, Bonetto C, Tosato S, Zanatta G, Cristofalo D, Salazzari D, Lazzarotto L, Bertani M, Bissoli S, De Santi K, Cremonese C (2014) First-contact incidence of psychosis in north-eastern Italy: influence of age, gender, immigration and socioeconomic deprivation. Br J Psychiatry 205(2):127-34.
12. Veling W, Susser E, Selten JP, Hoek HW (2015) Social disorganization of neighborhoods and incidence of psychotic disorders: a 7-year first-contact incidence study. Psychol Med 45(9):1789-98.
13. Hardoon S, Hayes JF, Blackburn R, Petersen I, Walters K, Nazareth I, Osborn DP (2013) Recording of severe mental illness in United Kingdom primary care, 2000–2010. PLoS One 8(12):e82365.
14. Tortelli A, Simon P, Lehouelleur S, Skurnik N, Richard JR, Baudin G, Ferchiou A, Leboyer M, Schürhoff F, Szöke A (2021) Characteristics associated with the risk of psychosis among immigrants and their descendants in France. Brain behavior 11(5):e02096.
15. Driessen G, Gunther N, Bak M, van Sambeek M, van Os J (1998) Characteristics of early-and late-diagnosed schizophrenia: implications for first-episode studies. Schizophr Res 33(1-2):27-34.
16. Fordham E, Gao CX, Filia K, O'Donoghue B, Smith C, Francey S, Rickwood D, Telford N, Thompson A, Brown E (2023) Social disadvantage in early psychosis and its effect on clinical presentation and service access, engagement and use. Psychiatry Res 328:115478.
17. Lee SC, DelPozo-Banos M, Lloyd K, Jones I, Walters JT, Owen MJ, O'Donovan M, John A (2020) Area deprivation, urbanicity, severe mental illness and social drift—A population-based linkage study using routinely collected primary and secondary care data. Schizophr Res 220:130-40.
18. Tibber MS, Kirkbride JB, Mutsatsa S, Harrison I, Barnes TR, Joyce EM, Huddy V (2019) Are socioenvironmental factors associated with psychotic symptoms in people with first-episode psychosis? A cross-sectional study of a West London clinical sample. BMJ Open 9(9):e030448.
19. Bosqui T, Shannon C, Anderson R, Turkington A, Barrett S, McCaul R, Cooper S, O’Donnell D, Rushe T, Mulholland C (2022) Neighbourhood effects on psychotic and depressive symptoms in the context of religious sectarianism in Northern Ireland: A data linkage study. Int J Soc Psychiatry 68(2):264-72.
20. Izquierdo A, Cabello M, Leal I, Torio I, Madrigal JL, MacDowell KS, Rodriguez-Jimenez R, Rentero D, Ibáñez Á, Ayora M, Díaz-Caneja CM (2023) Neighborhood vulnerability and disability in first episode of psychosis: a multilevel study. J Clin Psychiatry 84(2):46177.
21. Veru-Lesmes F, Rho A, Joober R, Iyer S, Malla A (2020) Socioeconomic deprivation and blood lipids in first-episode psychosis patients with minimal antipsychotic exposure: implications for cardiovascular risk. Schizophr Res 216:111-7.
22. Oher FJ, Demjaha A, Jackson D, Morgan C, Dazzan P, Morgan K, Boydell J, Doody GA, Murray RM, Bentall RP, Jones PB (2014) The effect of the environment on symptom dimensions in the first episode of psychosis: a multilevel study. Psychol Med 44(11):2419-30.
